# Evaluation of post-operative pain following dental procedures under local anesthesia among pediatric patients at Al Rass, Saudi Arabia

**DOI:** 10.6026/973206300200341

**Published:** 2024-04-30

**Authors:** Eyad M Alduwayghiri

**Affiliations:** 1 Department of Orthodontic and Pediatric Dentistry, College of Dentistry, Qassim University, Saudi Arabia

**Keywords:** Pain, discomfort, analgesic, pediatric dentistry

## Abstract

Evaluation of post-operative pain in children after receiving dental treatments under local anesthesia is of interest. Hence, a study consisting of 182 children aged from 4 to 12 years old, who received at least one of the following procedures: dental restoration, extraction, placement of stainless steel crown (SSC) with or without pulpotomy is completed. Parents were contacted by phone within 48 hours to assess post-operative pain by using Wong-Baker FACES Pain Rating Scale (WBF). Individuals who were reachable via phone (146 out of 182, response rate 80.2%). 30.8% of them reported experiencing pain or discomfort (WBF more than or equal 2). Pediatric patients who had SSC on their primary molars had a considerably higher incidence of reporting pain than any other dental procedures (44.8% at p < 0.001). However, there was no significant difference in reported pain between placements of SSC alone or SSC with pulpotomy (44.8% and 46.5%, respectively). Further, over-the-counter analgesics were administered in 19.9% of participants

## Background:

Treating pediatric patients can be challenging, especially when a lengthy course of treatment is needed. Furthermore, children receiving thorough oral treatment may experience many forms of physical and psychological damage. Numerous factors, such as the procedure's type and duration, can contribute to postoperative symptoms [1]. Pediatric dentists usually provide comprehensive dental treatment with their patients in a conscious state. However, some children suffer from extreme anxiety. Dental caries continues to be one of the most common health issues affecting children [2]. Following dental procedures, pain or discomfort are often experienced [3]. In general, pain is an extremely unpleasant experience that affects people of all ages whose sensory and cognitive systems are fully formed [4]. Misconceptions regarding the use of analgesics have resulted in under treatment of pain in all age groups [3]. It is believed that 65% of patients feel some level of pain following dental procedures [4]. Pain during pediatric dentistry procedures may cause a parent to stop providing care. Young children frequently lack the mental capacity to use pain control techniques. In many circumstances, pain is regarded as a subjective phenomenon that differs from person to person. Due to varying degrees of cognitive development, comprehension of the questions, reaction to pain, fear, and anxiety, children have trouble rating pain [4, 5]. The most reliable method for assessing pain is self-reporting. Children find it difficult to convey and define their discomfort, which makes it harder for them to perceive it [1]. The conventional strategy for assessing pain throughout any surgery is to measure each patient's individual degree of pain [4, 5]. Females were reported to report discomfort more frequently by Acs and Drazner, while no such association was identified by Staman *et al.* [3]. According to earlier research, children experience post-operative pain at frequency of 38 to 42.8% [6]. The American Pain Society and the American Academy of Pediatrics jointly released recommendations in 2001. According to one definition, pain is a subjective experience that results from the interaction of emotional and sensory elements in the setting of culture and environment [3]. It is common knowledge that in pediatric dentistry, painful treatment is a major contributing cause to dental panic; hence it is imperative to manage pain effectively during primary tooth extractions. More focus is needed on how to minimize pain and discomfort in pediatric dentistry clinics [7]. The application of pain measurement scales like the Wong-Baker FACES Pain Rating Scale - which consists of six cartoon faces with a range of expressions from extremely pleased to extremely sad- has received strong validation [3, 8]. The FPS-R and the WBF are the most popular and most validated facial pain scales for measuring children's self-reported pain intensity [7]. The majority of post-operative dental discomfort (PDP) has not been studied. A common response to complaints of discomfort is to give a medication that relieves the pain. Analgesic medication use has been rising over time to treat children's post-operative pain [9]. The use of these medications may be influenced by parental expectations for post-operative pain and analgesic usage practices [10]. Therefore, it is of interest to evaluate pain and use of analgesic agents in children following surgical and restorative dental procedures in primary molars treated under local anesthesia.

## Materials and Method:

The present observational prospective study was performed in AlR ass dental clinics, Qassim University, Saudi Arabia, during the period Feb 2021- April 2022. This study was approved by the Ethical Committee (DRC/011FA/32), College of Dentistry, and Qassim University. The research consisted of 182 children aged from 4 to 12 years old, who received at least one of the following dental procedures under local anesthesia: dental restoration, extraction, placement of stainless steel crown (SSC) with or without pulpotomy. All procedures performed by one pediatric dentist. Exclusion criteria included children under analgesic at the day of the appointment, any procedure done on permanent teeth, if the parents could not be reached by phone within 48 hours after the procedure, children requiring conscious sedation or general anesthesia and those unable to communicate verbally. Informed consent was obtained from a parent for each participant included in the study. A structured form was developed to gather information, including the patient's age, gender, tooth location, type of local anesthetic, dental treatment and materials used, and behavior via the Frankl Scale Rating Pain and discomfort were assessed with the Wong-Baker FACES Pain Rating Scale (Figure 1 - see PDF) using https://wongbakerfaces.org/. This scale consists of six cartoon faces with a range of expressions from "no hurt" (score=0) to "hurts worst" (score=10). Score 2 or more in the Scale considered pain or discomfort in this study. No prescription for pain medication was given. Another pediatric dentist, who was unaware of the treatment, attempted to contact each parent or guardian within 48 hours to assess pain and discomfort and if there is any complaints made by the child; the presence, severity, and duration of pain or discomfort; whether or not analgesics were administered; anything else that they would like to report. Pearson Chi-Square test was utilized to assess any relationships between the variables and post-operative pain or analgesic use.

## Results:

146 out of 182 with response rate 80.2% were successfully reached by phone and the remaining 36 participants were excluded. 59% males and 41% females ranging from 4 to 12 years old (mean age = 7.4 years old; median age = 7 years old), participated in the study.

All patients received local anesthetic (2% lidocaine with 1:100000 epinephrine ratio). At the time of the follow-up phone call after 48 hours dental procedure, 30.8% of the children reported pain or discomfort, as reflected by the Wong-Baker FACES Pain Rating Scale (Score 2 or more considered as feeling discomfort or pain). Table 1 shows post-operative pain and analgesic usage by type of procedure. Patients who had received stainless steel crown (SSC) only and those who had received a pulpotomy and SSC were significantly more likely to report discomfort (44.8% and 46.5%, respectively). The vast majority of them reported score 2 and 3 in the scale within 48 hours after dental procedure. 19.9% of the children were given over-the-counter analgesics by their parents to either prevent pain or because the child complained of pain. However, analgesics were used most when SSCs were placed (31%).

## Discussion:

More attention to the incidence of pain or discomfort following some of dental procedures in Pediatric Dentistry is needed. When receiving dental treatment on dental chair under local anesthesia, some of pediatric children feels anxious, which might make them more sensitive to discomfort or pain. Data is consistent with the results of Staman *et al.* when they found a significant difference in post-operative pain after placement of stainless steel crowns on primary molars. Children treated with stainless steel crowns had a much higher rate of post-operative pain among other types of dental procedures; it was 58% of the cases [3]. Also in our study, the treatment of primary molars with stainless steel crowns was strongly related with post-operative discomfort in 44.8% of the cases. When the treatment of stainless steel crowns were combined with pulpotomy, 46.5% of the pediatric patients reported discomfort. There was no significant difference in reported discomfort between placement of stainless steel crowns alone and placement of stainless steel crowns with pulpotomy. Following pulp therapy treatments, children sometimes experience some pain; the intensity of the pain varies depending on the level of pre-operative infection. Non vital teeth with an abscess demonstrated a higher prevalence of pain [11]. Askenazi *et al.* found similar results to these findings, patients undergoing SSCs with or without pulpotomies experienced a significantly higher incidence of pain compared to extractions, restorations, and sealants (P<0.001) [10]. With regards to post-operative pain after dental extraction, 6 out of 27 participants in this study who had received at least one primary molar extraction, reported discomfort or pain in Wong-Baker FACES Pain Rating Scale (22.2%), which was lower than what Baillargeau *et al.* found in their study (37.3%) [7].This is may be due to the limited extraction cases in this observational study Hu *et al.* conducted a study to determine the prevalence of postoperative dental morbidity in children as well as the variables linked to this type of morbidity. They calculated the pain score using the Wong-Baker FACES Pain Rating Scale. They came to the conclusion that postoperative dental pain was more common than bleeding. Parents noticed that their children were in more pain immediately following the procedure, as well as one and three days later. It was discovered that the number of teeth treated overall was correlated with postoperative dental pain. Compared to postoperative dental pain, postoperative bleeding was less common [11]. Our findings also found a significant difference in the use of analgesics following placement of SSC with or without pulpotomy (31% - 30.2%, respectively) while it was (14.8%) after extraction, and only (6.4%) after dental restorations. These findings shows the differences between the type of dental procedure and pain severity, were more common following placement of SSC [9]. Levin L. *et al.* assessed the frequency, duration, and usage of analgesics in people following dental procedures, taking into account factors such as gender, injection type, completed dental procedure, and dental history. They came to the conclusion that after deep restorations, there was a higher frequency of severe post-operative pain [14]. The limitation of the current study was restricted to smaller geographic location as well as information about preoperative analgesic use was not taken which might reduce the prevalence of postoperative pain and discomfort as reported by Primrosch *et al.* [15]. Further studies are needed to validate the findings and to minimize post-operative pain following dental procedures in pediatric patients.

## Conclusion:

Post-operative discomfort and pain occur most commonly after the placement of stainless steel crowns on primary molars. The pain incidence following placement of stainless steel crowns was higher than for dental extractions and dental restorations. Pediatric patients who had received stainless steel crowns (with or without pulpotomy) reported similar results of post-operative pain. One-fifth of the participants reported the use of over-the-counter analgesic medications. Further measures are needed to minimize post-operative pain following dental procedures in children.

## Figures and Tables

**Table 1:** Post-operative pain or discomfort and analgesic use following dental procedures

**Procedure**	**Participants**	**Average age (yrs)**	**Reporting pain within 48 hours after the procedure (%)**	**Taking medications (%)**
Dental restoration	47	7.3	6 (12.8%)	3 (6.4%)
Extraction	27	8.8	6 (22.2%)	4 (14.8%)
Stainless steel crowns (SSC)	29	7.1	13 (44.8%)	9 (31%)
Pulpotomy + SSC	43	6.5	20 (46.5%)	13 (30.2%)
	146	7.4	45 (30.8%)	29 (19.9%)
